# Efficient Tuning of the Opto-Electronic Properties of Sol–Gel-Synthesized Al-Doped Titania Nanoparticles for Perovskite Solar Cells and Functional Textiles

**DOI:** 10.3390/gels9020101

**Published:** 2023-01-24

**Authors:** Qana A. Alsulami, Zafar Arshad, Mumtaz Ali, S. Wageh

**Affiliations:** 1 Chemistry Department, Faculty of Science, King Abdulaziz University, Jeddah 21589, Saudi Arabia; 2 School of Engineering and Technology, National Textile University, Faisalabad 37640, Pakistan; 3 Department of Physics, Faculty of Science, King Abdulaziz University, Jeddah 21589, Saudi Arabia

**Keywords:** electron transport materials, metal doping, metal-oxide nanoparticles, sol–gel synthesis, photoactive textiles, electronic band-structure tuning

## Abstract

The efficient electron transport layer (ETL) plays a critical role in the performance of perovskites solar cells (PSCs). Ideally, an unobstructed network with smooth channels for electron flow is required, which is lacking in the pristine TiO_2_-based ETL. As a potential solution, here we tuned the structure of TiO_2_ via optimized heteroatom doping of Al. Different concentrations (1, 2, and 3 wt%) of Al were doped in TiO_2_ and were successfully applied as an ETL in PSC using spin coating. A significant difference in the structural, opto-electronic, chemical, and electrical characteristics was observed in Al-doped TiO_2_ structures. The opto-electronic properties revealed that Al doping shifted the absorption spectra toward the visible range. Pure titania possesses a bandgap of 3.38 eV; however, after 1, 2, and 3% Al doping, the bandgap was linearly reduced to 3.29, 3.25, and 3.18 eV, respectively. In addition, higher light transmission was observed for Al-doped TiO_2_, which was due to the scattering effects of the interconnected porous morphology of doped-TiO_2_. Al-doped titania shows higher thermal stability and a 28% lower weight loss and can be operated at higher temperatures compared to undoped titania (weight loss 30%) due to the formation of stable states after Al doping. In addition, Al-doped TiO_2_ showed significantly high conductivity, which provides smooth paths for electron transport. Thanks to the effective tuning of band structure and morphology of Al-doped TiO_2_, a significant improvement in current densities, fill factor, and efficiency was observed in PSCs. The combined effect of better Jsc and FF renders higher efficiencies in Al-doped TiO_2_, as 1, 2, and 3% Al-doped TiO_2_ showed 12.5, 14.1, and 13.6% efficiency, respectively. Compared to undoped TiO_2_ with an efficiency of 10.3%, the optimized 2% Al doping increased the efficiency up to 14.1%. In addition, Al-doped TiO_2_ also showed improvements in antibacterial effects, required for photoactive textiles.

## 1. Introduction

Nanoparticles offer unique characteristics depending on heteroatom doping, orientation, and morphology; which can be tuned according to the end application. Scientists are faced with designing electronics using the versatile properties in nanomaterials to meet energy demands [1]. There are several parameters to be considered regarding nanomaterial processing, such as enhanced surface area, tuned bandgap, and controlled electron mobility. Based on the distinctive features of nanomaterials, the performance of energy harvesting devices is being pushed over time. For instance, solid-state perovskite solar cells (PSCs) have emerged as a rising star in the world of photovoltaics to meet energy challenges [2,3]. Therefore, an exponential increase in the efficiency of PSCs has been recorded in the last decade. In PSCs, the electrons and holes are diffused from perovskite and then transported through the electron transport layer (ETL) and hole transport layer (HTL). The energy levels alignment is favorable for charge extraction and suitable conductivity results in the charge transport from the absorber layer to the external circuit [4]. This work focuses on the Al doping of TiO_2_ to achieve tuned opto-electronic characteristics for an efficient ETL of PSCs. In this way, the charge carriers are effectively separated and recombination can be suppressed for enhanced photoconversion efficiency [5].

Recently, Soek employed TiO_2_ as a mesoscopic scaffold layer in a PSC, where formamidinium lead iodide (FAPbI_3_) was used as an absorber material to achieve an efficiency of up to 20% [6]. TiO_2_ has an indirect and wide bandgap of 3.37 eV at room temperature and a high binding energy (60 meV), suitably required for ETM [7]. Snaith and Yang employed metal-doped TiO_2_ and obtained an efficiency of up to 12% after a tunable bandgap [8]. Other aspects of the ETL have also been extensively explored, including high stability, low-temperature processing, suitable Fermi levels, and hysteresis-free charge transport [9]. ETL engineering enables fast electron transportation by suppressing the hindrance to electron flow, hence the charge recombination-induced energy loss of electrons is impeded. Similarly, ETL thickness, porosity, and uniformity can optimize the electron flow and minimize the recombination rate [10]. Recently scientists also developed graphene/TiO_2_ and yttrium-doped TiO_2_ to fabricate a device at room temperature with long-term stability [11]. A few solar cell structures have also been prepared without an ETL; however, the resulting PCEs tend to be low compared to PSCs prepared with an ETL [12].

To date, various studies have been conducted to enhance the performance of PSC via the heteroatom doping of the ETL. In this regard, the alkaline-earth metal-doped TiO_2_ thin films are coated onto conductive glass by spin coating in PSCs [13]. TiO_2_ thin films with a dopant concentration of more than 5% are not feasible to prepare using the sol–gel method [14]. Lithium is another dopant for the TiO_2_-based ETL that can improve photovoltaic performance due to the elimination of interstitial defects [15]. The introduction of Li^+^ ions into the TiO_2_ lattice reduces interstitial defects, which gives rise to a higher electron conductivity without significantly altering the valence band edge [16]. Compared to noble metals, the earth-abundant nature of Al doping is advantageous for commercial prospects. Al is a highly corrosion-resistant and abundant metal in nature to be used as an N-type dopant in electron transport materials for perovskite solar cells [17]. Al-doped titania possesses suitable electronic properties and can be synthesized via facile synthesis methods. For photocatalytic applications, it shows a minor leaching effect and does not pose any biohazard in biological applications, unlike heavy metals such as Cd, Pb, etc. Al-doped TiO_2_ as an electron extraction material has been used in perovskite solar cells to significantly enhance the efficiency and other electrical parameters of the ETL [18]. Doped TiO_2_ possesses a smooth surface morphology and helps in reducing trap sites thereby reducing the band gap and enhancing the photovoltaic efficiency by up to 7.68%, compared to undoped (6.98%) [18]. Al-doped TiO_2_ mesoporous PSCs possess a high power conversion efficiency, suitable band gap, large absorption coefficient, long carrier diffusion length as well as an enhanced current density. The enhanced efficiency in Al-doped TiO_2_ is attributed to substitutional doping into the titania lattice by reducing defect densities and passivating electron trap states in bulk due to the stoichiometry of the metal oxide [19]. The effect of Al-doped titania on the fabrication and opto-electronic performance was reported by Effat et al. [20]. Different concentrations of Al doping were tested to optimize the optical band gap (around 3.3 eV) and electrical performance in PSCs (13.97%). Extending the same idea, here we explored the facile sol–gel synthesis of TiO_2_ particles doped with Al for enhanced photovoltaic and photoactive textiles.

Photoactive textiles are emerging with great scientific interests after COVID-19. Antimicrobial and antibacterial materials damage the cell wall and rupture the microbe, which results in the inhibition of growth pathways in microbes. Exciton is produced due to photon absorption in semiconductor materials, which generates reactive oxygen species (ROS) [21]. These ROS are responsible for the photocatalytic degradation of organic materials and microbes [22]. By coating the semiconductor materials on textile substrates, photoactive textiles can be fabricated [23]. In addition, such textiles are equally suitable for the degradation of organic pollutants from the water via photocatalysis. Passivation on porous textile substrates allows the easy recovery of active material after water treatment.

The Sol–gel method was chosen for its diverse and facile synthesis of versatile ceramic structures with high homogeneity. The structure and properties of the nanomaterials can be controlled by tuning pH, time, and low processing temperatures [24]. It also requires a low cost of the required equipment, while yielding high purity and homogeneity with mild reaction conditions. It could potentially be used for the synthesis of highly crystalline TiO_2_ of high-purity [25]. Defects, interfacial resistance, and morphology are the major contributors to the conversion efficiency of the ETL in PSCs. In this study pristine TiO_2_ nanoparticles synthesized via the sol–gel method showed low conversion efficiency compared to Al-doped TiO_2_, possibly due to the reduced defects and increased electronic conduction with doping. Additionally, the Al-doped TiO_2_ photoanode showed higher thermal stability, along with operating at higher temperatures. Additionally, we also tested Al-doped TiO_2_ photoanodes as antibacterial and photocatalytically active textile electrodes for dye degradation.

In this work, a sol–gel solution of TiO_2_ with 1, 2, 3 wt% Al was prepared and deposited onto a fluorine-doped Tin-oxide (FTO) glass substrate. Perovskite solar cells were fabricated with various dopant concentrations of Al. These thin films were then characterized via X-ray diffraction (XRD) and Raman spectroscopy to check the structural properties and to check the effect of the dopant on the TiO_2_ lattice. In addition, the surface morphology and opto-electronic properties were analyzed, which showed a significant effect of doping on the electronic structure. Additionally, enhancement in the electrical properties of the TiO_2_ films was calibrated via the Hall effect. The optimized doping (2%) of Al resulted in a significant enhancement of the current density (Jsc) and fill factor (FF), hence increasing the power conversion efficiency of the solar cells. Similarly, compared to the undoped TiO_2_, Al-doped TiO_2_ possessed better photoactivity.

## 2. Materials and PSC Fabrication

The main precursor for the synthesis of TiO_2_, including titanium isopropoxide (TTIP) (liquid of 97% purity), was procured from Sigma-Aldrich (Burlington, MA, USA). Aluminum isopropoxide (ATIP) powder of 99% purity was purchased from Acros Organics (Geel, Belgium). Absolute ethanol (C_2_H_6_O, ≥99.8%), monoethanolamine (C_2_H_7_NO), MEA 99% purity were purchased from Sigma-Aldrich. Nitric acid (68%) and NaOH were also purchased from Sigma-Aldrich. All chemicals were of analytical grade and were used as received (without any further purification). Conductive fluorine-doped tin oxide (FTO)-glass (TEC 8, Pellington Co., Baltimore, MD, USA) was used for the fabrications of the PSCs.

To synthesize the undoped and Al-doped TiO_2_, weight ratios of TTIP and ATIP were measured as Ti_1−x_Al_x_O (where x = 0.01, 0.02, and 0.03). This weight ratio of Al precursor was slowly dissolved in 20 mL TTIP along with 20 mL ethanol, 80 mL de-ionized water, and 0.1 M NaOH. It was stirred at 450 rpm at 60 °C for 3 h, to achieve homogeneous mixing. Afterward, 2 mL nitric acid was added for gel formation, which was then stored at room temperature for 12 h. The synthesis process for trace Al-doped TiO_2_ gels was the same as pristine TiO_2_, except for the addition of ATIP with various doping content during sol–gel synthesis.

**Figure 1:**
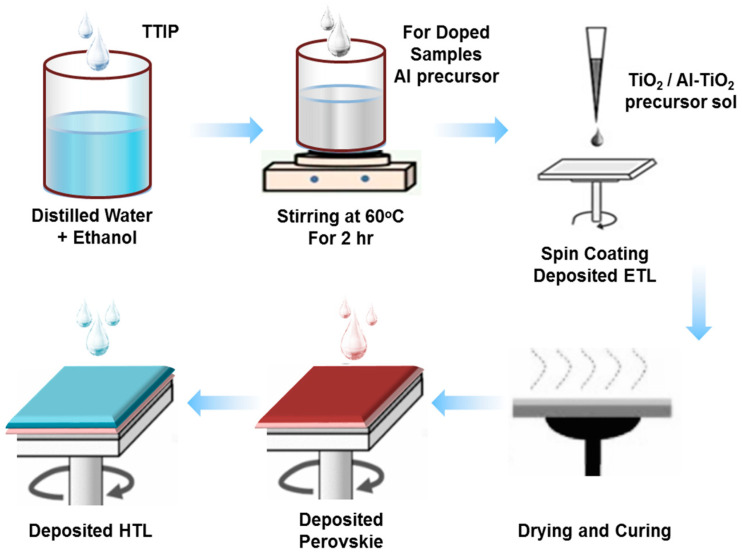
Schematic illustration of the sol–gel synthesis process of TiO_2_ nanoparticles and its application as an ETL in a PSC device.

FTO glass was pre-cleaned in an ultrasonic bath in the presence of 10 mL ethanol and 40 mL distilled water for 30 min. It was allowed to dry under an air stream and then subjected to ultraviolet ozone treatment for 20 min. After this cleaning, organic materials present on the surface of the substrate were completely removed. For undoped ETL layers, 5 mL undoped TiO_2_ (40–50 nm average particle size) was coated using a spin coater on the FTO substrate. A spin coater with optimized 1500 rpm and pressure 1 torr for 30 s was adopted for uniform surface coverage of the substrate. It was cycled 7 times for dense dispersion and satisfactory adhesion to reduce crack possibilities [26]. For Al-doped TiO_2_ films, it was allowed to spin coat at the same spin rate to obtain 1 wt%, 2 wt%, and 3 wt% Al-doped TiO_2_ thin films. All the thin films were first dried in an oven at 50 °C, with ambient atmospheric conditions for 10–15 min and after annealed at 450 °C for 2 h. Then, for the deposition of the perovskite absorber layer, 20 μL of lead bromide (PbBr_2_, 0.2 mM), 10 μL formamidinium iodide (FAI, 1 mM) 10 μL methyl ammonium bromide (MABr, 0.2 mM) and PbI_2_ (1.1 mM) were dissolved in dimethyl formamide (DMF, 0.8 mL) and 0.2 mL dimethyl sulfoxide (DMSO, 0.2 mL). It was all spin-coated on ETL thin films at 4500 rpm for 7 s. For crystallization of the perovskite layer, 90 μL of anhydrous chlorobenzene was dropped on the spinning substrate and dried on a hot plate for 15 min at 100 °C [27]. Finally, for deposition of the HTL layer (350 nm), spiro-OMeTAD (100 mg) and chlorobenzene (1.094 mL) were spin-coated (without dopants) on the absorber layer at 2000 rpm for 20 s. The perovskite layer and HTL were both fabricated under an inert atmosphere in the glove box. Silver back contacts were applied for effective charge transportation with sputtering, under 2 torr pressure for 30 s. Deposition of the silver back contacts provides circuit-aligned electron conduction in the perovskite solar cells. The scan rate for the current–voltage curves was 0.5 V s^−1^ [28]. The synthesis process is demonstrated in Figure 1.

## 3. Characterization

The crystal structure of different undoped and Al-doped nanoparticles were revealed by XRD diffraction in a 2θ range of 10 to 80°. The scanning speed was 2°/min at an acceleration voltage of 40 kV. The X-ray source Cu Kα manufactured by Rigaku Denki (Rigaku-D/MAX2500, Tokyo, Japan) was used, equipped with a characteristic wavelength of 1.5410 Å. JAD programming was then used to identify peaks. To support the results of XRD, Raman spectra were further studied to explore the structure using Raman spectroscopy (NWS-3100, JASCO, Tokyo, Japan), with an excitation wavelength of 532 nm. The surface morphologies of undoped and Al-doped nanoparticles were observed using scanning electron microscopy (SEM), instrument model: JSM-6490A JEOL (Tokyo, Japan). UV–visible spectroscopy (Shimadzu Co, Kyoto, Japan) was performed for analysis of light absorption, transmittance attributes, and band gap. A Tauc plot was then used to obtain the band gaps for all un-doped and Al-doped samples. A Fourier transform infrared (FTIR) spectrophotometer was used in an attenuated total reflectance (ATR) mode for functional group analysis (Manufacturer; Thermo Fisher Scientific Inc., Model; Nicolette iSTM 10). The infrared spectra was recorded in the range of 4000 cm^−1^ to 650 cm^−1^, with a goal of 2 cm^−1^. For the thermal stability test, TGA was performed on a TGA 5500 (TA Instruments) under a nitrogen flow rate of 35 mL min^−1^ for 30 min. The 10 mg sample of each powder sample was heated to 900 °C for around 42 min till the mass was stabilized and the organic matter was oxidized. The Brunauer–Emmett–Teller (BET) surface area of the films was measured using a Quantachrome Instruments NOVA4000, from the adsorption curve of the nitrogen isotherm. The photoelectrochemical characteristics and the electrochemical impedance spectroscopy (EIS) measurements of the PSCs were recorded with a potentiostat/galvanostat (PGSTAT 30, Autolab, EcoChemie, Utrecht, the Netherlands) under 100 mW cm^−2^. The frequency range was explored from 10 mHz to 65 kHz. For electrical measurements of the ETL, Hall effect measurements were taken using a swin system of 5300 G magnetic field under dark conditions and at a temperature of 300 K. For solar cell characteristics, efficiency and current density values were determined by a solar light simulator (Newport 94043A, Irvine, CA, USA) with a power of AM 1.5 simulated lights (100 mWcm^−2^). 

Antibacterial measurements were taken by *Escherichia coli* (*E.coli*) and *Staphylococcus aureus* (*S. aureus*) standard AATCC 147. This was a qualitative test where a single colony of *S. aureus* bacteria was introduced into a broth. This was preheated at 121 °C for 15 min and incubated at 37 °C for 24 h. This culture was rotated at 150 rpm for sculpture growth, and then placed into a Petri dish for bacterial inoculum after serial dilution. Finally, samples were place under UV light for 30 min and then into the bacterial lawn for 24 h at 37 °C. For photocatalysis measurements, Al-doped TiO_2_ nanoparticle-coated fabric was immersed in methylene blue (MB) dye solution, to determine their photocatalytic efficacy under sunlight. Photocatalysis was performed under sunlight with irradiance determined using a lux meter. The MB dye solution with a concentration of 10 ppm was used for both tests. To approximate the photocatalytic activity, the difference in maximum absorption intensity of the MB at 664 nm was recorded using a UV–visible spectrometer.

## 4. Results and Discussion 

### Structural and Morphological Characteristics

**Figure 2:**
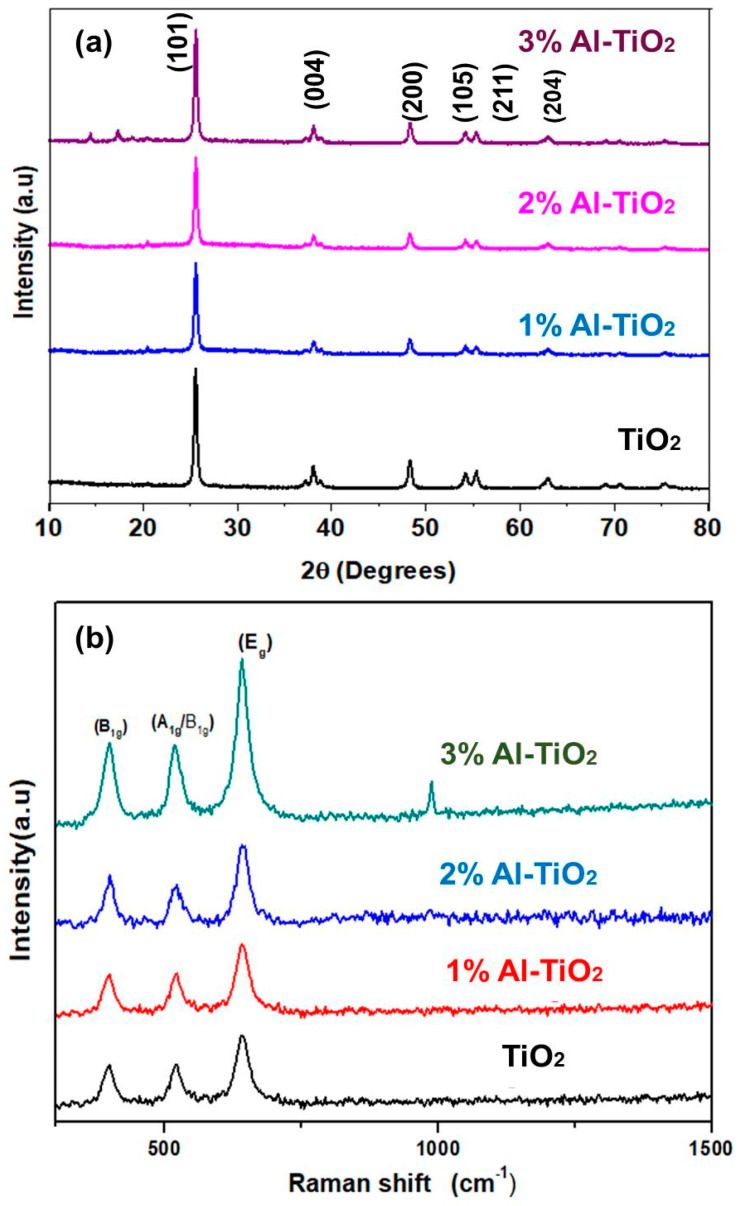
(**a**) XRD spectra of TiO_2_, 1% Al-TiO_2_, 2% Al-TiO_2_ and 3% Al-TiO_2_, (**b**) Raman spectra of TiO_2_, 1% Al-TiO_2_, 2% Al-TiO_2_ and 3% Al-TiO_2_.

XRD analysis was performed to better understand the effect of Al doping on the crystal structures of TiO_2_ nanoparticles. XRD spectra for all calcined undoped and 1, 2, and 3 wt% Al-doped TiO_2_ thin films are shown in Figure 2a. XRD showed the anatase phase of TiO_2_, with peaks positioned at angles (2θ) of 25.28, 37.8, 48.02, 53.89, 55.06, and 62.6°, associated with the planes (101), (004), (200), (105), (211), and (204), respectively. These peaks correspond with anatase TiO_2_, as evaluated from the JCP (21-1272) [29]. The anatase phase of TiO_2_ was not disturbed in all the doping concentrations, as shown in the spectra. It reveals that Al doping does not produce any separate crystal phase and Al is completely substituted into the doped samples without affecting their inherent structures. Up to 2% concentration, an impurity peak was not detected; however, at a higher concentration (3%), additional impurity peaks related to Al-O were detected in the 15–18° range. It is important to note that the intensity of impurity peaks is negligible, which shows that the major content of TiO_2_ exists in that structure. 

Overall, after the addition of the dopant, all peaks were of high-intensity and sharp, showing that the crystalline nature was retained. As we increased the dopant concentration (up to 2%) in the TiO_2_ lattice, peak intensity for the (101) and (200) planes was increased, hence preferential growth of these planes occurs in the presence of the Al-dopant [30]. Additionally, the peaks positioned at (101) widened after doping, related to perturbation in the anatase crystal of TiO_2_. With 2% Al doping, the anatase phase was not affected; however, at 3% doping, the crystallinity and structure of TiO_2_ was slightly disturbed. These minor shifting of the peaks is because of the random orientations of the dopant. The average crystallite size, strain, and dislocation density measurements were calculated using full-width half maximum (FWHM), as reported in Table 1. Crystallite size decreased from 24 nm to 18 nm after the addition of the dopant, thus concluding that Al doping has a strong effect on the thin-film properties. However, subtle expansion in the FWHM was observed due to Al doping. Dislocation density also increased in the same manner because of the random orientations of the ETL crystals, which support the transportation of electrons [31]. 

In order to determine the vibrational modes and to further probe the crystal structure of the nanoparticles, Raman scattering was studied. Figure 2b shows the Raman spectra of the undoped and 1–3% Al-TiO_2_ to support the XRD analysis in the range of 200–1500 cm^−1^. There are three dominant peaks of the Raman spectra, positioned at 434.5, 522–530, and 630–680 cm^−1^ [32]. The peaks at 434.5 cm^−1^ originate from the B_1g_ mode of both TiO_2_ and Al-doped TiO_2_. These are the polar bonds of O-O in the titania lattice, whose intensity is increased after Al doping, because of a higher resonance. The peak around 522 cm^−1^ is the phono mode of TiO_2_, which becomes right-shifted and wider after the addition of Al because of the tensile stresses generated by Al doping. The peak in the range 630–680 cm^−1^ (Eg mode) indicates O-Ti-O vibrations in the sub-domains of the crystallites, which showed a similar decrease in the size observed in XRD [33]. As Al (up to 2%) is present in minor quantities there was no significant changes in peak positions after doping; however, at higher concentrations (3%) an Al-O-related impurity peak was observed around 1050 cm^−1^. From both the XRD and Raman spectra, it can be concluded that above 2% concentration the Al doping causes the impurity peak, thus weakening the intrinsic TiO_2_ structure. 

**Figure 3:**
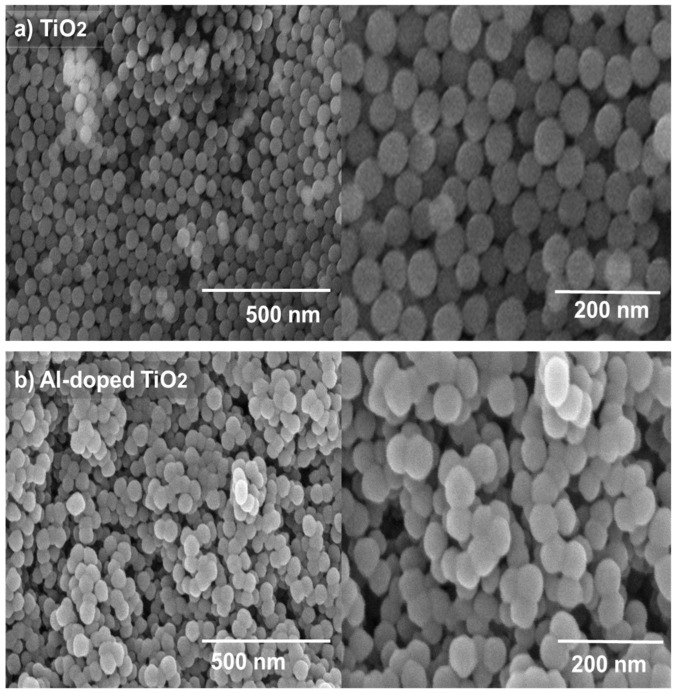
SEM analysis of (**a**) undoped TiO_2_, and (**b**) Al-doped TiO_2_ films coated onto FTO glass. Images were taken at low (**left**) and high (**right**) resolution.

Morphology of the ETL thin film was studied to understand the surface properties, defect analysis, and homogeneity of the film. The SEM morphology of the ETL thin films of TiO_2_ and Al-doped TiO_2_ (2% doping) is shown in Figure 3a,b. SEM analysis revealed a random assembly of particles in the form of a nanonetwork, with a uniform circular morphology. TiO_2_ nanoparticles were uniformly dispersed onto the FTO substrate, without cracks or agglomeration, as shown in Figure 3a. At a higher resolution (Figure 3a right) TiO_2_ particles showed an average diameter of 50 nm, dispersed onto the glass surface with a finely connected nanonetwork. It was observed that the overall diameter of the individual-doped particles was in the range of 40–50 nm, clearly observed in the high-resolution image. Such an interconnected network provides a suitable interface for the electron injection from the absorber layer and flow to the FTO glass. The densely packed surface coverage of the nanoparticle films increases the efficiency because the photoelectrons can be efficiently transferred to the external circuit via the FTO glass [34]. The undoped TiO_2_ films seem dense, as commonly employed for the photoanode in the perovskite solar cell. 

Figure 3b shows SEM image of a 2% Al-doped TiO_2_ structure obtained after a 450 °C annealing temperature. Calcined Al-doped TiO_2_ showed fused interconnected particles, with a large surface area and a relatively higher porosity than undoped TiO_2_. Stronger interconnections of particles provide a smooth path for electron flow, thus positively influencing the current density of the PSCs [35]. After Al doping, interconnected linkages among the particles were observed, which supports the conduction of photoelectrons through the ETL. The Al ions induce the preferential one-dimensional growth, hence causing the formation of a spontaneously fused particle structure. The individual-doped particles possess diameter of 45–50 nm, as is clear in the high-resolution image of Figure 3b right side. The doped particles showed a slightly fused structure; offering suitable connections, uniform size distribution, and a circular morphology. In addition, the aggregation during the film formation of such a fused nanoparticle structure is less, yielding a porous film structure compared to the undoped particles.

**Figure 4:**
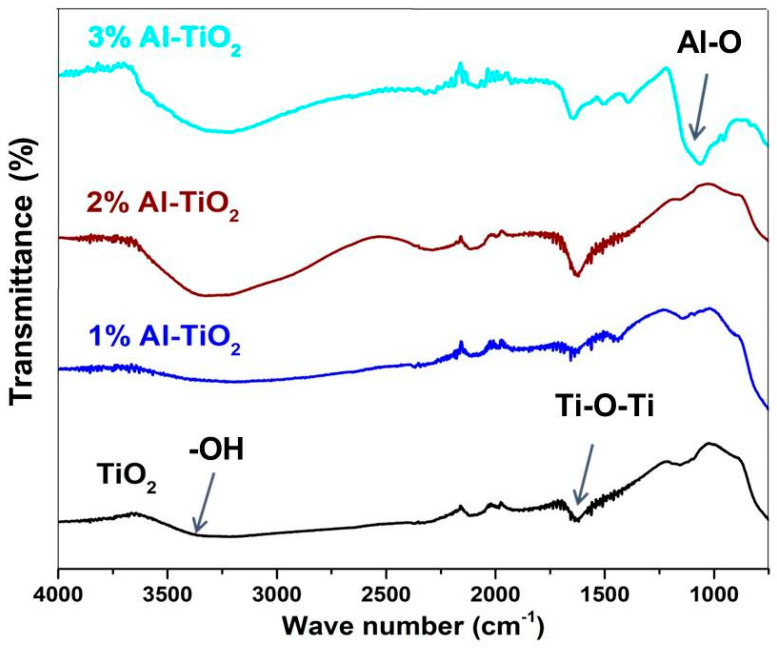
FTIR spectra of undoped TiO_2_, and 1−3% Al-doped TiO_2_.

FTIR spectroscopy was carried out to evaluate the variation in the functional groups upon doping with different concentrations of Al. These functional groups affect the interface formed with the absorber layer and also affect the electronic structure. FTIR spectra were recorded in ATR mode and the percentage difference was detected in transmittance, in the range of 500 to 4000 cm^−1^, as shown in Figure 4. The vibrational mode peak present around 3000 to 3650 cm^−1^ is attributed to the hydroxyl functional groups, which are present on the surface of TiO_2_. By Al doping of up to 2%, the hydroxyl content increased because of the higher dangling bonds on the surface and the higher surface area (discussed in next section). Whereas 3% Al doping again reduced the hydroxyl content due to the formation of Al-O impurity crystals, hence the dangling bonds were reduced [36]. These hydrophilic functional groups induce dispersion stability of the particles and a uniform film formation, due to the electrostatic dispersion stability provided by the hydroxyl functional groups. The surface hydroxyl content is directly proportional to the photocatalytic activity due to the better interface with the dye molecules. Additionally, the hydroxyl groups can behave as a center for capturing the photo-excited electrons, thereby enhancing the dwell time of the exciton. As the electron transport layer is embedded inside the absorber layer, the stability of the solar cell will not be significantly affected. The peak around 1600 cm^−1^ is associated with Ti-O-Ti bonds, hence observed in all the samples. In 3% Al-TiO_2_, a low-frequency peak around 1100 cm^−1^ is the characteristic peak for Al-O bonds, whose intensity is significant at the 3% concentration. With 2% doping, the Al-O functional groups did not increase significantly, so its related peak was not detected. Al doping ions induce a surface charge and a higher content of dangling bonds on the surface, hence increasing the hydroxyl content [37]. Similar characteristic spectra after Al doping has also been previously observed [38].

**Figure 5:**
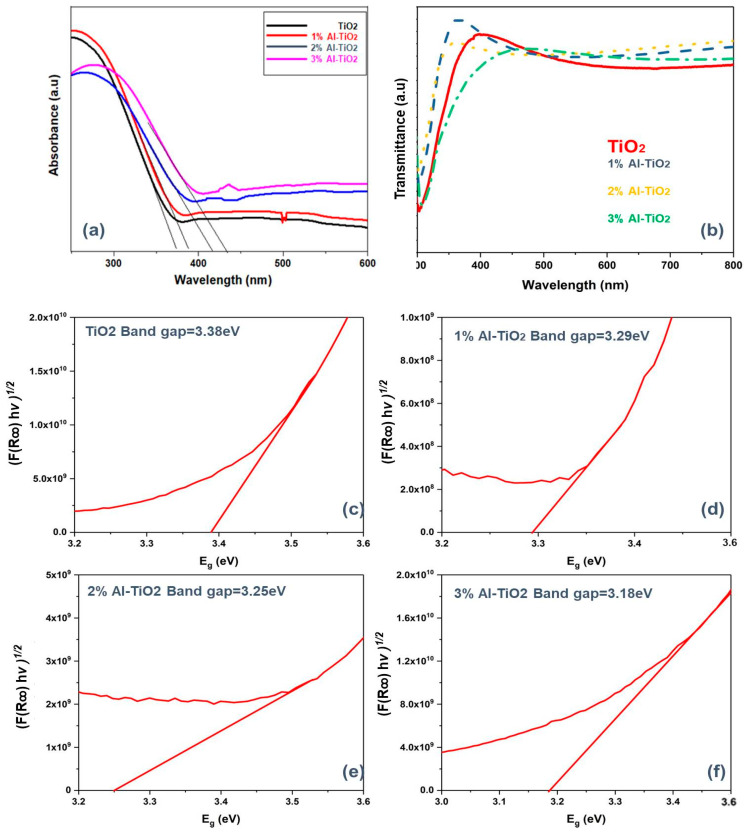
(**a**) Absorption spectra of 0–3% Al-doped TiO_2_. (**b**) Transmittance spectra of 0–3% Al-doped TiO_2_. Band gap calculated using a Tauc plot, for (**c**) undoped TiO_2_, (**d**) 1% Al-TiO_2_, (**e**) 2% Al-TiO_2_, and (**f**) 3% Al-TiO_2_.

In order to investigate the differences in opto-electronic properties by various Al-doping concentrations on TiO_2_; UV–Vis spectroscopy was carried out. The absorbance was measured for the colloidal solution of nano-particles; however, transmittance was characterized in the solid-state by casting a thin film of nanoparticles on the FTO glass. The Optical absorption spectra and transmittance spectra were recorded between 200 to 800 nm, as shown in Figure 5a. Both pristine and doped TiO_2_ showed a selective high-energy absorption range, a specific absorption in the deep UV and near UV regions. As undoped TiO_2_ possesses a high bandgap, a strong absorption was only observed in the ultraviolet range. On the other hand, this absorption range was slightly shifted toward the visible spectrum after Al doping. This extended visible spectrum absorption is related to the lowering of the bandgap, as Al doping generates low-energy states in the band structure of pristine TiO_2_ (details discussed in the next section) [39]. 

Despite having a higher absorbance (in colloidal form), the overall transmittance of the Al-doped TiO_2_ (in solid thin film form) was higher (Figure 5b). Specifically, the transmittance of the high-energy spectrum was also high, which is due to the scattering effects and porous structure of the Al-doped TiO_2_, as discussed in the SEM section. Better transmission of light renders higher photoexcitation in the absorber layer, hence offering better light-harvesting characteristics [40]. The bandgap for the undoped TiO_2_ and Al-doped TiO_2_ was calculated by the indirect band gap Equation (1) from the UV–Vis absorbance as shown in Figure 5c–f, which is the X-intercept of the Tauc plot.

(1) (αhv)1/n=A[(hv−Eg)]

Pure titania possesses a bandgap of 3.38 eV; however, after 1, 2, and 3% Al doping, the bandgap linearly reduced to 3.29, 3.25, and 3.18 eV, respectively. The shift in the absorbance range shows a considerable edge shift towards the visible range, which implies a reduction in the bandgap due to the narrowing of the bandgap energy by Al doping. This decreased bandgap after Al doping is consistent with the findings of previous reports [41]. The reduction in the bandgap is due to the Burstein–Moss effect, which means that the Fermi levels are decreased due to the formation of intermediate states between the valence bands and conduction bands. These intermediate states support the conduction of photoelectrons from the valence band towards the conduction band at lower energies, thereby increasing its photo-absorbance range. This bandgap tuning results in a significant decrease in the bandgap of 3.38 to 3.18 eV, hence it allows electron transitions to an excited state with lower energies. Moreover, Al doping allows the recombination of electrons between the valence and conduction bands [42]. The higher content of hydroxyl groups observed in the FTIR spectra—formed after Al doping—also improves the dwell time. Charge carriers are separated by hydroxyl groups and oxygen vacancies contributing toward a lower recombination, resulting in improved current densities and efficiencies of the PSCs [43]. 

**Figure 6:**
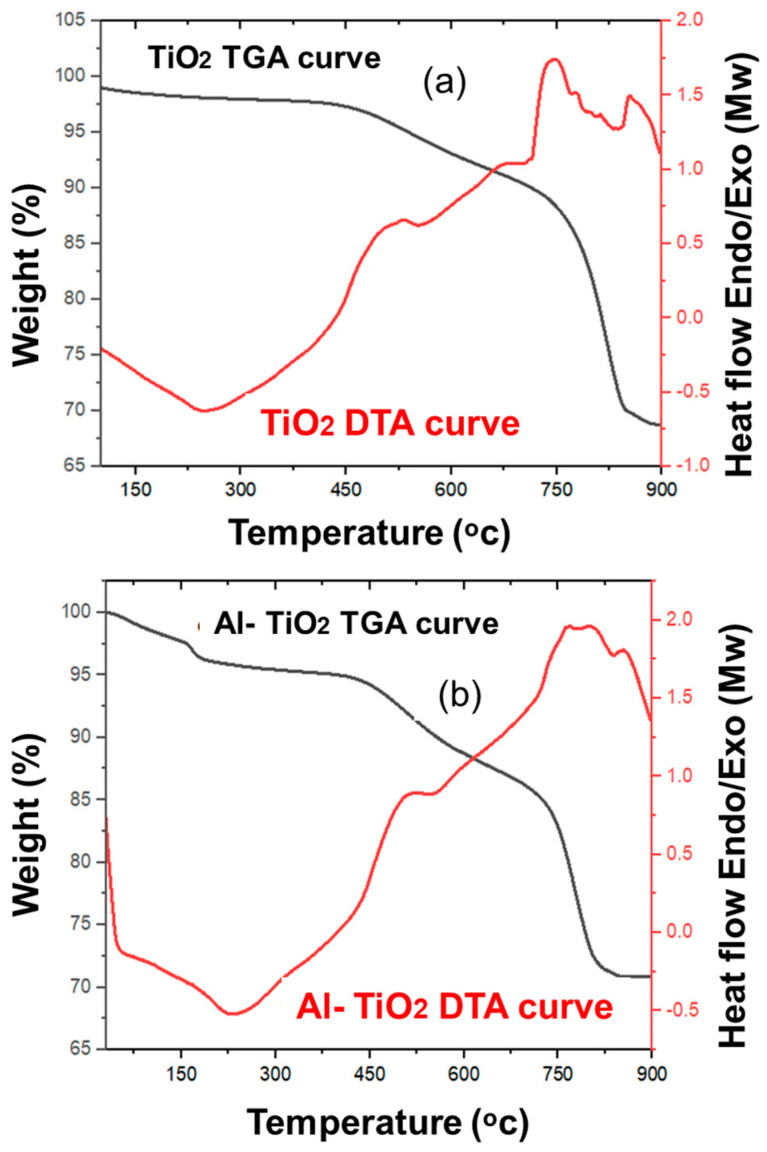
(**a**) TGA/DTA of TiO_2_, and (**b**) TGA/DTA of Al-doped TiO_2_.

TGA/DTA curves of undoped TiO_2_ and Al-doped TiO_2_ are reported in Figure 6, to study the thermal stability of the solar cell. From the TGA curves for undoped TiO_2_, weight loss is majorly categorized into three different zones for clarity. First, weight loss falling in the range of 0–125 °C, related to moisture removal. Second, weight loss around 125–400 °C due to the decomposition of amorphous carbon produced from monoethanol amine (used as a gelation agent during sol preparation) and hydroxyl group removal in this range [1]. While the third zone in the range of 400–790 °C is due to severe structural changes, such as the conversion of the rutile phase of TiO_2_ into the anatase phase [2]. These groups were not detected in the FTIR spectroscopy because FTIR is performed for calcined nanoparticles. In a given spectra, there is a 30% weight loss in the case of pristine titania; however, there is 28% weight loss when Al is doped into titania. Al-doped titania offers higher thermal stability and can be operated at higher temperatures compared to undoped titania. Alumina is doped into titania because of the removal of the hydroxyl and organic reagents in the structure [32]. Figure 6b depicts the initial weight loss curve at almost 150 °C; it is, of course, due to the decomposition of trapped ethanol and deionized water from the pores. The second weight loss curve ranges from 450 to 800, this curve indicates the structural changes due to the dopant material and the conversion of titania. After this, the weight loss between 480 to 830 °C is due to conversion of the rutile phase titania into the anatase phase and also the removal of amine and other groups used as the titania precursors. Al doping into TiO_2_ increases the crystallinity; therefore, it is stable at high temperatures and weight loss is minimal. The XRD results also demonstrates that particle size varied with 3 wt% dopant meaning structural changes occurred. Whereas weight loss was negligible after 800 °C. These thermal characterizations are potentially applicable in dye-sensitized solar cells where a repeated thermal annealing process is carried out. 

In the DTA curves there are two exothermic reaction peaks in the undoped and doped graphs. The exothermic peaks of the undoped appeared at 750 °C, while the Al-doped graph peaks appeared at 730 °C. Here, the heat absorption process is observed due to structural changes. The appearance of earlier peaks for Al/TiO_2_ is due to the incorporation of Al into the titania lattice. There are endothermic reaction peaks in both graphs. In the case of the undoped titania an endothermic peak is formed at 225 °C. Whereas doping Al causes an earlier endothermic peak at 210 °C. At this stage, a phase change occurs, and heat is released for the doped titania due to the incorporation of Al which promotes the crystalline phase change. These results are consistent with the XRD results showing the crystalline to amorphous phase transition. In pristine TiO_2_ a greater amount of heat is required, so its phase transition mainly occurs at higher temperatures; however, Al-doped TiO_2_ requires less energy. 

**Figure 7:**
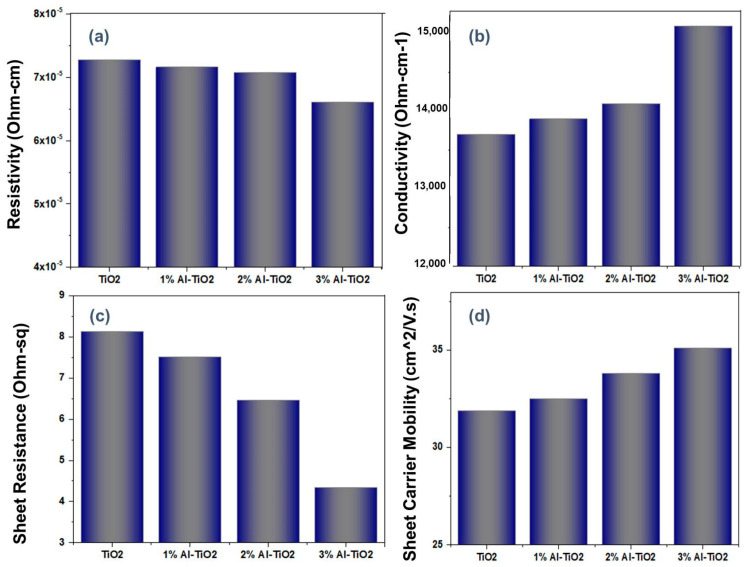
Comparison of the (**a**) resistivity, (**b**) conductivity, (**c**) sheet resistance, and (**d**) carrier mobility of pristine TiO_2_ and Al−doped TiO_2_.

Electrical measurements of the doped and undoped ETLs were performed using Hall measurements to check the electron transport characteristics of the ETLs in the PSCs. Electrical parameters, i.e., conductivity, resistivity, sheet resistance, and sheet carrier mobility were recorded and shown in Figure 7a–d. The thickness of the photoanodes was measured using an optical profilometer followed by electrical characterizations. The thicknesses of the undoped and doped titania thin films were approximately 3 µm to 3.5 µm. As all samples were fabricated under the same conditions, there was no significant difference in the thicknesses of the thin films. Resistivity is the hindrance to the flow of electrons along the conductive path. Pure TiO_2_ showed a resistivity of 7.28 × 10^−5^ Ohm/cm, upon Al doping the resistivity decreased by up to 6.61 × 10^−5^ Ohm/cm (Figure 7a). When Al is doped into titania, defects are reduced, and a smooth path for electrons conduction is available. As Al-doped TiO_2_ has a fused-particle structure, less confinement and a shorter path for charge flow is available [44]. Conductivity is inversely proportional to resistivity, so the conductivity of the ETLs is increased from 1.37 × 10^4^ to 1.51 × 10^4^ Ohm.cm^−1^ (Figure 7b). Conductivity was increased with Al doping, which is also due to the development of the crystalline structure [45]. At lower concentrations, Al is interstitially doped due to its relatively small size. Upon oxidation Al-O is formed; however, separate particles of Al-O were not observed. This electron-rich electronic structure favors the flow of electrons, thus increasing the conductivity and electron transport characteristics. Al incorporation into TiO_2_ increases the concentration of charge carriers. A significant decrease in the sheet resistivity and an increase in carrier mobility was found with the Al doping, as is clear from Figure 7c,d. Undoped TiO_2_ showed an electron mobility of 3.19 × 10^1^ cm^2^/V·s, which increased to 3.25 × 10^1^, 3.38 × 10^1^, 3.51 × 10^1^ cm^2^/V.s for 1, 2, and 3% Al-doped TiO_2_, respectively. An inverse relation for the sheet resistivity was observed for Al doping. Amalina et al. [46] suggested that the energy states formed by Al doping are of lower energy, hence a reduction in the bandgap and increased sheet carrier mobility were observed. Here, better conductivity can potentially provide better charge transport characteristics in the TiO_2_ films.

**Figure 8:**
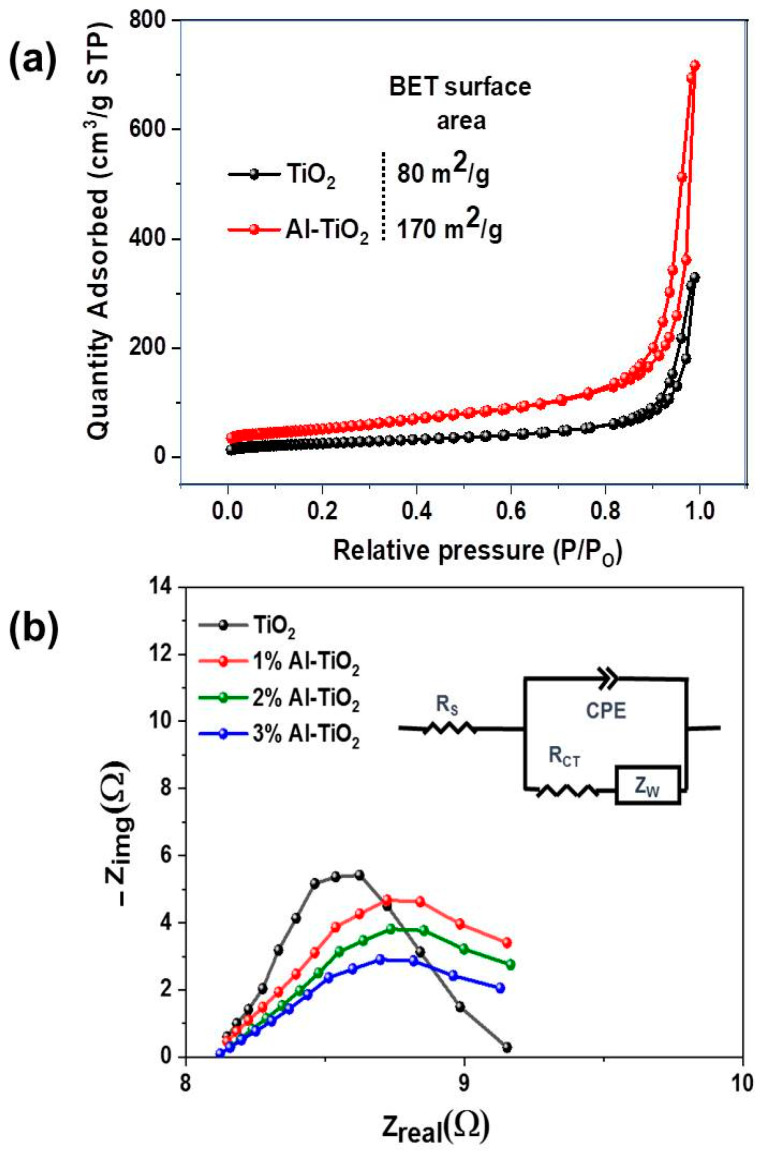
(**a**) Nitrogen adsorption–desorption isotherms of TiO_2_ and Al-TiO_2_. (**b**) EIS spectra of TiO_2_, 1% Al-TiO_2_, 2% Al-TiO_2_ and 3% Al-TiO_2_.

The specific surface area of the powders was tested using a Braunet–Emit–Teller analysis, as shown in Figure 8a. The specific surface area of the powder was calculated from the adsorption curve of the isotherm. Pristine TiO_2_ showed a relatively low specific surface area of 80 m^2^/g, due to the larger size and lower functionality of particles causing aggregation in the powder form. On the other hand, the smaller size and higher functionality of the Al-doped TiO_2_ offered a higher surface area of 170 m^2^/g. High surface areas also enhance the transmission of light through the thin film in transmission mode UV–visible absorbance. Additionally, a higher surface area is responsible for higher adsorption of dye in photocatalysis, as observed under the initial dark conditions. A higher surface area offers better catalysis as more light is entrapped and available for catalysis. It can be observed that Al doping significantly enhances the surface area without affecting pore size and results in a higher lifetime of generated electrons. 

To understand the charge transfer behavior of the solar cells, an electrochemical impedance spectrum (EIS) was performed. These measurements were performed for all samples at ambient temperature as shown in Figure 8b. The charge transfer dynamics were calculated using a dummy cell structure, where both photoanodes were sandwiched face-to-face, using 60 µm Surlyn. This dummy cell was filled with iodide electrolytes to avoid the contribution of other components in the EIS and only check the resistance contribution of the ETL, the EIS was performed in an ETM/electrolyte/ETM structure used in our previous work [47]. Using a Nyquist plot and Randles fitting model (Figure 8b: inset), it was concluded that the increase in Al content causes a decrease in the charge transfer resistance, i.e., height of the Nyquist curve. However, there was no significant difference in series resistance, i.e., X-intercept of the EIS curve. If the charge transfer resistance value is low, there will be an effective moment of electrons across the junction. Pristine titania possesses a higher resistance of 5.18 Ohm compared to the Al-doped titania showing 2.6 Ohm (3% Al doping). A significant decrease in resistance is because of the stable structure formed on the surface via doping, which is favorable for charge transportation. The series resistance of the EIS curve is mainly dependent on substrate conductivity; which is FTO in all the cases, thus a similar series resistance was observed. 

**Figure 9:**
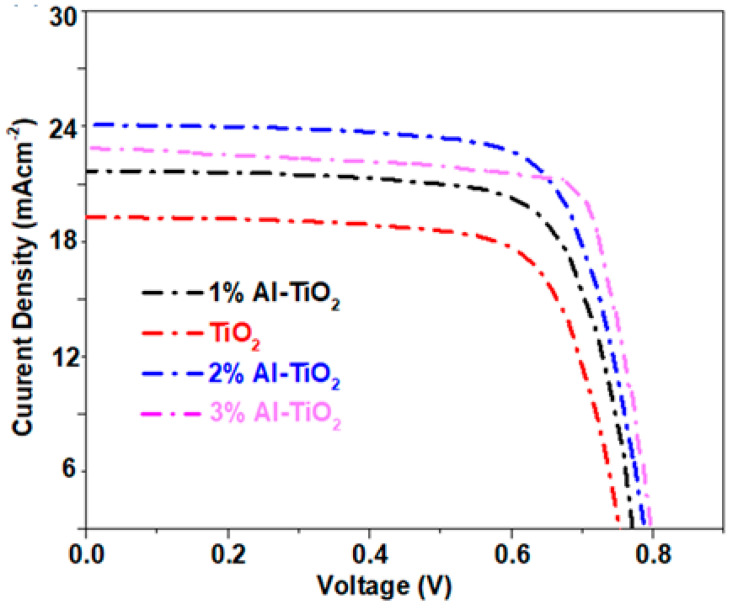
J-V characteristics of the PSCs based on the undoped TiO_2_, 1% Al-TiO_2_, 2% Al-TiO_2_, and 3% Al-TiO_2_.

In order to unravel the charge transport dynamics after introducing the Al dopant in the ETL, a J-V analysis of the fabricated PSCs was carried out. The PSCs fabricated using undoped TiO_2_, and 1, 2, and 3 wt% Al-doped TiO_2_ displayed a significant difference in performance, as shown in Figure 9a. From this data, the difference in the short circuit current density (Jsc), fill factor (FF), and open-circuit voltages (V_oc_) of the PSCs resulted in a variation in the power conversion efficiency, as summarized in Table 2. 

Al doping affects the Jsc more prominently compared to other factors. For instance, the Jsc for pristine TiO_2_ is 19.51 mA/cm^2^; however, it reached a maximum at 24.58 mA after 2% Al doping. This is because that with a 2% Al doping the conduction of electrons is improved without compromising the transmittance. However, at a 3% Al-doping concentration a significant portion of the visible light spectrum is absorbed by the ETL itself. As 2% Al was incorporated into the TiO_2_, the presence of Al increases the charge mobility, hence suppressing the charge recombination. In addition, the tuning of the Fermi level and conduction band also favors quenching electrons from the perovskite layer to TiO_2_ [48]. Band alignment with Al-doped TiO_2_ provides efficient ETLs that aggress the electron transport, along with suppressing the recombination rates. The SEM structure also revealed that Al-doped nanoparticles are interconnected, which favors electron transport through longer paths. Better light transmission through the porous structure formed by Al-doped TiO_2_ also plays a critical role in enhancing the Jsc [49]. The transparent nature of the 2% Al-doped TiO_2_ causes better light absorption by the perovskite layer, thereby increasing the Jsc. On the other hand, in the 3% Al doping, Jsc is again decreased to 23.13 mA/cm^2^ due to the blockage of light by the ETL itself. As a lower content of light reaches the perovskite layer, the Jsc is, therefore, reduced in the 3% Al-doped sample. Moreover, Al doping significantly increases the FF by increasing the conductivity of the TiO_2_. The internal resistance of the solar cells is reduced after Al doping, thereby increasing the FF. The combined effects of the better Jsc and FF renders higher efficiencies in the Al-doped TiO_2_, as 1, 2, and 3% Al-doped TiO_2_ showed 12.5, 14.1, and 13.6% efficiencies, respectively. Compared to the undoped TiO_2_ with an efficiency of 10.3%, the optimized 2% Al doping increased the efficiency by 14.1%. In conclusion, with the facile doping of Al, a significant performance improvement was achieved.

Amongst all the photovoltaic parameters, the difference in the V_OC_ was insignificant. For instance, the undoped TiO_2_ showed a V_oc_ of 0.75 V, increased to 0.77 V for the optimized sample. This minor difference could be related to the lower potential loss in the Al-doped TiO_2_ and slightly better band alignment of the Al-doped TiO_2_. Compared to the optimized (2%) doping, a significant increase in the V_oc_ (6.6%) was observed in 3% Al doping. This might be due to the inter-bridging of the Jsc and Voc. As the conductivity of 3% Al doping is significantly high, it can transport less electrons (as Jsc is low) with negligible energy loss, so V_oc_ is improved in this case. 

**Figure 10:**
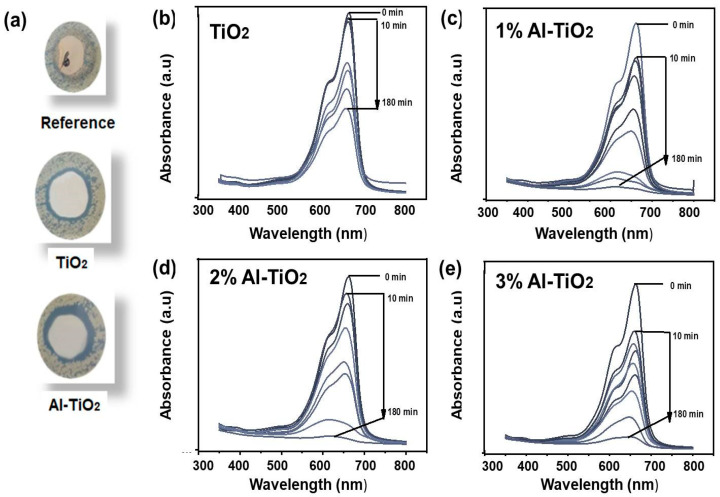
(**a**) Qualitative antibacterial measurements for the undoped and Al-doped TiO_2_. Photocatalytic dye degradation measurements for (**b**) TiO_2_, (**c**) 1% Al-TiO_2_, (**d**) 2% Al-TiO_2_, (**e**) 3% Al-TiO_2_.

Photoactive textiles are demanding due to their potential against disease spreading, especially after the COVID-19 breakout [50,51]. For this purpose undoped and Al-doped titania were applied onto fabric by a dip–dry–cure method, after making a 2% solution in an acrylate binder. The antibacterial activity of the fabrics treated with the undoped and Al-doped TiO_2_ was investigated, as shown in Figure 10a. The antibacterial characterization revealed that the Al-doped TiO_2_ possessed an enhanced antibacterial activity compared to the undoped TiO_2_. The zone of inhibition formed by the Al-doped TiO_2_ was 3.05 mm, whereas the zone of inhibition formed by the pristine TiO_2_ was 2.32 mm. The Al-doped TiO_2_ possessed a greater zone of inhibition due to the stronger photocatalytic effect of the Al-doped TiO_2_ nanoparticles. As a result of photoabsorption, ROS were generated, which have a strong destructive potential against bacteria [52]. A stronger photocatalytic activity produces a higher vicinity around the active material; hence the growth of bacteria in that vicinity was restricted. Therefore, the Al-doped TiO_2_ exhibited a larger inhibition zone and a stronger potential to resist bacteria. The reference cotton sample showed no inhibition zone and hence the growth of bacteria on the untreated cotton fabric, as can be observed in Figure 10a.

The Al-doped TiO_2_ nanoparticles were coated onto fabrics by a dip–dry–cure method, after making a 2% solution in an acrylate binder. The photocatalytic activity of the fabrics treated with the undoped and Al-doped TiO_2_ was investigated, as shown in Figure 10b–e. Their reaction pace against the MB dye in an aqueous solution was measured to determine their photocatalytic efficacy. We conducted the degradation experiment in a double-layer quartz tube container, similar to an immersion photoreactor. The MB dye solution with a concentration of 10 ppm was used for the dye degradation test. The maximum absorption intensity of MB was observed at 664 nm; in the absorption spectra recorded using a UV–visible spectrometer. After adding a 4 × 4 cm coated fabric sample to 50 mL of dye solution, the mixture was agitated continuously for 30 min at room temperature in the dark in order to determine the adsorption/desorption equilibrium between the catalyst and dye. After, we subjected the stable aqueous dye solution to visible light irradiation for various time intervals, recording the UV absorption spectra, continuing this process for a total of 180 min (values taken in the intervals of 10, 20, 40, 60, 80, 150, and 180 min). To determine MB degradation in the presence of light, Equation (2) was used:

(2) $$D\%=\frac{1-C_{t}}{C_{0}}\times 100$$

where *C*_0_ is the concentration of the dye solution at the beginning of the photocatalytic process and *C_t_* is the concentration of the dye solution at the measurement time [53]. The photodegradation of the dyes using the pristine and Al-doped electrodes was calculated after the saturation of adsorption, and the samples were exposed to light for photocatalytic dye degradation. Comparing the photocatalysis of all electrodes, it can be observed that the undoped TiO_2_ electrode degraded 20% of the original concentration, whereas the 1% Al-TiO_2_ electrode degraded 95%. While the 2% and 3% Al-TiO_2_ electrodes showed a degradation of 92% and 85%, respectively. In addition, the metallic centers in the semiconductors serve as an electron sink, thanks to their conductive nature. This effect led to better charge separation and a greater dwell time for photocatalysis. Mobile phase (powder form) photocatalysts offer higher activity; however, after passivating the photocatalysts on the fabrics, the performance was drastically low, as the catalytic reaction is diffusion controlled. This is related to the lower surface area intact with the active material and organic pollutant, and the diffusion-controlled reaction dynamics. This significant improvement in photocatalytic degradation of our proposed electrode showed that doping is strongly related to an increase in photocatalytic activity. However, excessive doping (3%) results in the formation of conductive centers, which serve as a recombination site for the exciton [54]. However, the easier recovery of active materials with minimal secondary pollution caused by the active materials themselves makes the proposed treatment system closer to practical application [55]. 

## 5. Conclusions

Here, we explored the potential of Al doping in trace quantities into TiO_2_, synthesized using the sol–gel method for the ETL in PSCs. The structural characterizations revealed that Al can be successfully doped into anatase TiO_2_ at different concentrations. Up to 2% concentration offers interstitial doping, without the formation of an impurity peak. However, at 3% and above, an impurity peak and significant blockage of the visible spectrum were observed; therefore 2% Al doping was considered optimal in this work. A 2% Al-doped TiO_2_ showed a fused-particle structure, making a thin film with a highly porous network. Pure titania possessed a bandgap of 3.38 eV; however, after 1, 2, and 3% Al doping the bandgap was linearly reduced to 3.29, 3.25, and 3.18 eV, respectively. The shift in the absorbance range showed a considerable edge shift towards the visible range, which implies that the reduction in band gap due to the narrowing of the band gap energy was by Al doping. The Hall effect characteristics showed an increase in conduction and electron mobility due to the addition of extra electrons in the electronic system. The J-V results showed that Al doping significantly improved the power conversion efficiency of the PSCs compared to the undoped titania-based PSCs. The efficiency of the undoped and doped TiO_2_ (2% Al doping) was recorded at 10.3% and 14.1%, respectively. In addition, the zone of inhibition formed by the Al-doped TiO_2_ was 3.05 mm, whereas the zone of inhibition formed by the pristine TiO_2_ was 2.32 mm. The Al-doped TiO_2_ possessed a greater zone of inhibition due to the stronger photocatalytic effect of the Al-doped TiO_2_ nanoparticles. The photodegradation of the dyes using the pristine titania and the Al-doped titania was 48% and 95% in 180 min, respectively. The significantly improved photodegradation of the dyes using the Al-doped titania is related to its higher light absorbance, generating higher excitons to participate in the photodegradation. 

## Figures and Tables

**Table 1:** FWHM, diffraction angle, and crystallite size calculations for all ETL samples.

Sample	Diffraction Angle 2θ (°)	FWHM	Crystallite Size D (nm)	Dislocation Density δ (10^14^ lines/m^2^)
TiO_2_	25.48	0.354	24	15.8
1% Al-TiO_2_	25.48	0.393	21	23.1
2% Al-TiO_2_	25.48	0.413	19	25.8
3% Al-TiO_2_	25.48	0.433	18	29.3

**Table 2:** J-V curve parameters for the TiO_2_-based ETL devices.

PSC	V_oc_ (V)	Jsc (mA)	FF	η (%)
Pristine TiO_2_ PSC	0.75	19.51	0.71	10.38
1% Al-TiO_2_ PSC	0.76	22.29	0.74	12.54
2% Al-TiO_2_ PSC	0.79	24.58	0.75	14.53
3% Al-TiO_2_ PSC	0.80	23.13	0.74	13.69

## References

[1] Tan H., Jain A., Voznyy O., Lan X., García de Arquer F.P., Fan J.Z., Quintero-Bermudez R., Yuan M., Zhang B., Zhao Y. (2017). Efficient and stable solution-processed planar perovskite solar cells via contact passivation. Science.

[2] Yang D., Zhou X., Yang R., Yang Z., Yu W., Wang X., Li C., Liu S.F., Chang R.P. (2016). Surface optimization to eliminate hysteresis for record efficiency planar perovskite solar cells. Energy Environ. Sci..

[3] Bai S., Da P., Li C., Wang Z., Yuan Z., Fu F., Kawecki M., Liu X., Sakai N., Wang J.T.-W. (2019). Planar perovskite solar cells with long-term stability using ionic liquid additives. Nature.

[4] Wang P., Li R., Chen B., Hou F., Zhang J., Zhao Y., Zhang X. (2020). Gradient energy alignment engineering for planar perovskite solar cells with efficiency over 23%. Adv. Mater..

[5] Wang K., Olthof S., Subhani W.S., Jiang X., Cao Y., Duan L., Wang H., Du M., Liu S.F. (2020). Novel inorganic electron transport layers for planar perovskite solar cells: Progress and prospective. Nano Energy.

[6] Kim H.-S., Park N.-G. (2014). Parameters affecting I–V hysteresis of CH3NH3PbI3 perovskite solar cells: Effects of perovskite crystal size and mesoporous TiO_2_ layer. J. Phys. Chem. Lett..

[7] Wu Y., Yang X., Chen H., Zhang K., Qin C., Liu J., Peng W., Islam A., Bi E., Ye F. (2014). Highly compact TiO_2_ layer for efficient hole-blocking in perovskite solar cells. Appl. Phys. Express.

[8] Hu W., Yang S., Yang S. (2020). Surface modification of TiO_2_ for perovskite solar cells. Trends Chem..

[9] Lindblad R., Bi D., Park B.-w., Oscarsson J., Gorgoi M., Siegbahn H., Odelius M., Johansson E.M., Rensmo H. (2014). Electronic structure of TiO_2_/CH3NH3PbI3 perovskite solar cell interfaces. J. Phys. Chem. Lett..

[10] Cojocaru L., Uchida S., Sanehira Y., Nakazaki J., Kubo T., Segawa H. (2015). Surface treatment of the compact TiO_2_ layer for efficient planar heterojunction perovskite solar cells. Chem. Lett..

[11] Deng X., Wang Y., Chen Y., Cui Z., Shi C. (2019). Yttrium-doped TiO_2_ compact layers for efficient perovskite solar cells. J. Solid State Chem..

[12] Liu D., Yang J., Kelly T.L. (2014). Compact layer free perovskite solar cells with 13.5% efficiency. J. Am. Chem. Soc..

[13] Wang J., Qin M., Tao H., Ke W., Chen Z., Wan J., Qin P., Xiong L., Lei H., Yu H. (2015). Performance enhancement of perovskite solar cells with Mg-doped TiO_2_ compact film as the hole-blocking layer. Appl. Phys. Lett..

[14] Baktash A., Amiri O., Sasani A. (2016). Improve efficiency of perovskite solar cells by using magnesium doped ZnO and TiO_2_ compact layers. Superlattices Microstruct..

[15] Teimouri R., Heydari Z., Ghaziani M.P., Madani M., Abdy H., Kolahdouz M., Asl-Soleimani E. (2020). Synthesizing Li doped TiO_2_ electron transport layers for highly efficient planar perovskite solar cell. Superlattices Microstruct..

[16] Hou X., Zhou J., Huang S., Ou-Yang W., Pan L., Chen X. (2017). Efficient quasi-mesoscopic perovskite solar cells using Li-doped hierarchical TiO_2_ as scaffold of scattered distribution. Chem. Eng. J..

[17] Abdelmouleh M., Boufi S., Belgacem M.N., Dufresne A. (2007). Short natural-fibre reinforced polyethylene and natural rubber composites: Effect of silane coupling agents and fibres loading. Compos. Sci. Technol..

[18] Kim J.Y., Rhee S., Lee H., An K., Biswas S., Lee Y., Shim J.W., Lee C., Kim H. (2020). Universal Elaboration of Al-Doped TiO_2_ as an Electron Extraction Layer in Inorganic–Organic Hybrid Perovskite and Organic Solar Cells. Adv. Mater. Interfaces.

[19] Pathak S.K., Abate A., Ruckdeschel P., Roose B., Gödel K.C., Vaynzof Y., Santhala A., Watanabe S.I., Hollman D.J., Noel N. (2014). Performance and stability enhancement of dye-sensitized and perovskite solar cells by Al doping of TiO_2_. Adv. Funct. Mater..

[20] Moshfeghi E., Entezari M.H. (2022). Enhancement of the photovoltaic performance of perovskite solar cells via sono-synthesis of Al-doped TiO_2_ as the electron transport layer. Int. J. Energy Res..

[21] Riaz R., Ali M., Anwer H., Ko M.J., Jeong S.H. (2019). Highly porous self-assembly of nitrogen-doped graphene quantum dots over reduced graphene sheets for photo-electrocatalytic electrode. J. Colloid Interface Sci..

[22] Riaz R., Ali M., Sahito I.A., Arbab A.A., Maiyalagan T., Anjum A.S., Ko M.J., Jeong S.H. (2019). Self-assembled nitrogen-doped graphene quantum dots (N-GQDs) over graphene sheets for superb electro-photocatalytic activity. Appl. Surf. Sci..

[23] Riaz R., Ali M., Maiyalagan T., Arbab A.A., Anjum A.S., Lee S., Ko M.J., Jeong S.H. (2020). Activated charcoal and reduced graphene sheets composite structure for highly electro-catalytically active counter electrode material and water treatment. Int. J. Hydrog. Energy.

[24] Najafi A., Sharifi F., Mesgari-Abbasi S., Khalaj G. (2022). Influence of pH and temperature parameters on the sol-gel synthesis process of meso porous ZrC nanopowder. Ceram. Int..

[25] Rahmani-Azad M., Najafi A., Rahmani-Azad N., Khalaj G. (2022). Improvement of ZrB2 nanopowder synthesis by sol-gel method via zirconium alkoxide/boric acid precursors. J. Sol-Gel Sci. Technol..

[26] Wang S., Liu B., Zhu Y., Ma Z., Liu B., Miao X., Ma R., Wang C. (2018). Enhanced performance of TiO_2_-based perovskite solar cells with Ru-doped TiO_2_ electron transport layer. Sol. Energy.

[27] Wang B., Ma J., Li Z., Chen G., Gu Q., Chen S., Zhang Y., Song Y., Chen J., Pi X. (2022). Bioinspired molecules design for bilateral synergistic passivation in buried interfaces of planar perovskite solar cells. Nano Res..

[28] Sun P., Qu G., Hu Q., Ma Y., Liu H., Xu Z.-X., Huang Z. (2022). Highly Efficient Large-Area Flexible Perovskite Solar Cells Containing Tin Oxide Vertical Nanopillars without Oxygen Vacancies. ACS Appl. Energy Mater..

[29] Sauvage F., Di Fonzo F., Li Bassi A., Casari C.S., Russo V., Divitini G., Ducati C., Bottani C.E., Comte P., Graetzel M. (2010). Hierarchical TiO_2_ photoanode for dye-sensitized solar cells. Nano Lett..

[30] Zhang Y., Liu X., Li P., Duan Y., Hu X., Li F., Song Y. (2019). Dopamine-crosslinked TiO_2_/perovskite layer for efficient and photostable perovskite solar cells under full spectral continuous illumination. Nano Energy.

[31] Wang W., Zhang Z., Cai Y., Chen J., Wang J., Huang R., Lu X., Gao X., Shui L., Wu S. (2016). Enhanced performance of CH3NH3PbI3− x Cl x perovskite solar cells by CH3NH3I modification of TiO_2_-perovskite layer interface. Nanoscale Res. Lett..

[32] Ilie A.G., Scarisoareanu M., Morjan I., Dutu E., Badiceanu M., Mihailescu I. (2017). Principal component analysis of Raman spectra for TiO_2_ nanoparticle characterization. Appl. Surf. Sci..

[33] Venkatasubbu G.D., Ramakrishnan V., Sasirekha V., Ramasamy S., Kumar J. (2014). Influence of particle size on the phonon confinement of TiO_2_ nanoparticles. J. Exp. Nanosci..

[34] Haider A.J., AL–Anbari R.H., Kadhim G.R., Salame C.T. (2017). Exploring potential environmental applications of TiO_2_ nanoparticles. Energy Procedia.

[35] Seo S., Shin S., Kim E., Jeong S., Park N.-G., Shin H. (2021). Amorphous TiO_2_ coatings stabilize perovskite solar cells. ACS Energy Lett..

[36] Kim D.I., Lee J.W., Jeong R.H., Yu J.-H., Yang J.W., Nam S.-H., Boo J.-H. (2019). Enhancing the optical properties using hemisphere TiO_2_ photonic crystal as the electron acceptor for perovskite solar cell. Appl. Surf. Sci.

[37] Yoo G.Y., Azmi R., Kim C., Kim W., Min B.K., Jang S.-Y., Do Y.R. (2019). Stable and colorful perovskite solar cells using a nonperiodic SiO_2_/TiO_2_ multi-nanolayer filter. ACS Nano.

[38] Chen S.-H., Chan S.-H., Lin Y.-T., Wu M.-C. (2019). Enhanced power conversion efficiency of perovskite solar cells based on mesoscopic Ag-doped TiO_2_ electron transport layer. Appl. Surf. Sci..

[39] Singh R., Dutta S. (2018). Synthesis and characterization of solar photoactive TiO_2_ nanoparticles with enhanced structural and optical properties. Adv. Powder Technol..

[40] Lee J.E., Oh S.-M., Park D.-W. (2004). Synthesis of nano-sized Al doped TiO_2_ powders using thermal plasma. Thin Solid Films.

[41] Muna Muzahim Abbas M.R. (2020). Solid State Reaction Synthesis and Characterization of Aluminum Doped Titanium Dioxide Nanomaterials. J. Southwest Jiaotong Univ..

[42] Kumar S., Verma N.K., Singla M.L. (2013). Study on reflectivity and photostability of Al-doped TiO_2_ nanoparticles and their reflectors. J. Mater. Res..

[43] Lin S.-S., Wu D.-K. (2009). Enhanced optical properties of Al-doped TiO_2_ thin films in oxygen or nitrogen atmosphere. Appl. Surf. Sci..

[44] Valaski R., Arantes C., Senna C., Carôzo V., Achete C., Cremona M. (2014). Enhancement of open-circuit voltage on organic photovoltaic devices by Al-doped TiO_2_ modifying layer produced by sol–gel method. Thin Solid Films.

[45] Khan M., Mehmood B., Mustafa G.M., Humaiyoun K., Alwadai N., Almuqrin A.H., Albalawi H., Iqbal M. (2021). Effect of silver (Ag) ions irradiation on the structural, optical and photovoltaic properties of Mn doped TiO_2_ thin films based dye sensitized solar cells. Ceram. Int..

[46] Liu J., Yang H., Tan W., Zhou X., Lin Y. (2010). Photovoltaic performance improvement of dye-sensitized solar cells based on tantalum-doped TiO_2_ thin films. Electrochim. Acta.

[47] Ali M., Anjum A.S., Bibi A., Wageh S., Sun K.C., Jeong S.H. (2022). Gradient heating-induced bi-phase synthesis of carbon quantum dots (CQDs) on graphene-coated carbon cloth for efficient photoelectrocatalysis. Carbon.

[48] Saravanan S., Dubey R. (2020). Study of Al-Doped and Al/N Co-Doped TiO_2_ Nanoparticles for Dye Sensitized Solar Cells. J. Mater. Environ. Sci..

[49] Murashkina A.A., Murzin P.D., Rudakova A.V., Ryabchuk V.K., Emeline A.V., Bahnemann D.W. (2015). Influence of the dopant concentration on the photocatalytic activity: Al-doped TiO_2_. J. Phys. Chem. C.

[50] Zhuo J. (2016). Photoactive Chemicals for Antimicrobial Textiles. Antimicrobial Textiles.

[51] Gao Z., Liu P., Fu X., Xu L., Zuo Y., Zhang B., Sun X., Peng H. (2019). Flexible self-powered textile formed by bridging photoactive and electrochemically active fiber electrodes. J. Mater. Chem. A.

[52] Mahltig B., Miao H. (2017). Microwave-assisted preparation of photoactive TiO_2_ on textile substrates. J. Coat. Technol. Res..

[53] Baylan E., Yildirim O.A. (2019). Highly efficient photocatalytic activity of stable manganese-doped zinc oxide (Mn: ZnO) nanofibers via electrospinning method. Mater. Sci. Semicond. Process..

[54] Lee S., Noh J.H., Han H.S., Yim D.K., Kim D.H., Lee J.-K., Kim J.Y., Jung H.S., Hong K.S. (2009). Nb-doped TiO_2_: A new compact layer material for TiO_2_ dye-sensitized solar cells. J. Phys. Chem. C.

[55] Ismael M. (2020). A review and recent advances in solar-to-hydrogen energy conversion based on photocatalytic water splitting over doped-TiO_2_ nanoparticles. Sol. Energy.

